# Proton-induced DNA damage promotes integration of foreign plasmid DNA into human genome

**DOI:** 10.3389/fonc.2022.928545

**Published:** 2022-09-02

**Authors:** Meghri Katerji, Antonella Bertucci, Valery Filippov, Marcelo Vazquez, Xin Chen, Penelope J. Duerksen-Hughes

**Affiliations:** ^1^ Department of Basic Sciences, Loma Linda University School of Medicine, Loma Linda, CA, United States; ^2^ Department of Radiation Medicine, Loma Linda University Medical Center, Loma Linda, CA, United States; ^3^ Center for Genomics, Loma Linda University School of Medicine, Loma Linda, CA, United States

**Keywords:** ionizing radiation, DNA damage, DNA integration, carcinogenesis, human papillomaviruses, reactive oxygen species

## Abstract

High-risk human papillomaviruses (HPVs) cause virtually all cervical cancer cases and are also associated with other types of anogenital and oropharyngeal cancers. Normally, HPV exists as a circular episomal DNA in the infected cell. However, in some instances, it integrates into the human genome in such a way as to enable increased expression of viral oncogenes, thereby leading to carcinogenesis. Since viral integration requires breaks in both viral and human genomes, DNA damage likely plays a key role in this critical process. One potentially significant source of DNA damage is exposure to elevated doses of ionizing radiation. Natural background radiation is ubiquitous; however, some populations, including radiological workers, radiotherapy patients, and astronauts, are exposed to significantly higher radiation doses, as well as to different types of radiation such as particle radiation. We hypothesize that ionizing radiation-induced DNA damage facilitates the integration of HPV into the human genome, increasing the risk of developing HPV-related cancers in the exposed population. To test this, we first determined the kinetics of DNA damage in keratinocytes exposed to ionizing radiation (protons) by assessing γ-H2AX foci formation using immunofluorescence (direct damage), and also measured ROS and 8-oxoG levels *via* DCFDA and Avidin-FITC (indirect damage).As anticipated, direct DNA damage was observed promptly, within 30 min, whereas indirect DNA damage was delayed due to the time required for ROS to accumulate and cause oxidative damage. Although radiation was lethal at high doses, we were able to establish an experimental system where radiation exposure (protons and X-rays) induced DNA damage dose-dependently without causing major cytotoxic effects as assessed by several cytotoxicity assays. Most importantly, we explored the impact of radiation exposure on integration frequency using a clonogenic assay and demonstrated that as predicted, proton-induced DNA damage promotes the integration of HPV-like foreign DNA in oral keratinocytes. Overall, the insights gained from this work enable us to better understand the contribution of radiation exposure and DNA damage to HPV-mediated carcinogenesis and direct us toward strategies aimed at preventing malignancies in HPV-infected individuals.

## Introduction

Human papillomavirus (HPV) is the most common sexually transmitted infection. According to the Centers for Disease Control and Prevention, 79 million Americans are currently infected with HPV, with another 20 million new infections occurring each year (1). In about 90% of these cases, the infection is cleared by the immune system within two years. However, a relatively small subset of infections persists, and of these, some progress to malignancy (2–4). High-risk HPVs are the causative agents of virtually all cases of cervical cancer as well as a significant percentage of other anogenital and oropharyngeal cancers. In fact, current estimates indicate that HPV infection may be associated with as many as 93% of anal cancers, 63% of oropharyngeal cancers, 40% of penile cancers, 64% of vaginal cancers, and 51% of vulvar cancers (5). It is projected that 5.2% of all cancers worldwide can be attributed to HPV (6, 7).

The oncogenic properties of high-risk HPVs are encoded in two viral oncoproteins, E6 and E7. The E6 oncoprotein forms a complex with p53 and targets it to proteasomal degradation, resulting in evasion of apoptosis and perturbation of cell cycle control. On the other hand, the E7 oncoprotein binds to Rb and causes its degradation, leading to the inappropriate release of E2F transcription factor which stimulates unrestrained replication and cell division. Therefore, when these viral oncoproteins are over-expressed, the HPV-infected cells undergo uncontrolled cell proliferation and survival, and consequently develop HPV-induced malignancies (7–9).

Normally, the HPV genome is present inside the host cells in the circular episomal form. Under these circumstances, the viral E2 gene regulates viral transcription and genome replication, and thereby maintains the expression of the viral E6 and E7 oncogenes at low levels, insufficient to cause malignancy (10, 11). In the course of carcinogenesis, however, the extrachromosomal viral genome often becomes integrated into the host genome. This integration event functions as a critical biological driver of cellular transformation, since it typically results in the disruption and loss of the negative regulator E2, allowing persistent over-expression of the E6 and E7 oncogenes (12–15). Less commonly, elevated expression of the viral oncogenes can also be achieved by genetic or epigenetic modifications of the HPV genome or the presence of an increased number of episomal HPV copies (16–19). While a high episomal viral load, combined with an absence of HPV integration, is frequently detected in precancerous lesions, a high proportion of invasive HPV-associated cancers contain the viral DNA integrated into the host genome (18, 20). Analysis of samples from The Cancer Genome Atlas study indicates that HPV integration occurs in >80% of HPV-positive cervical cancers. Of these, 76% of HPV-16 positive samples contained integrated HPV, whereas integration was detected in all HPV18-positive samples (21, 22). The rate of HPV integration in other anogenital cancers is not as well documented, with one study reporting that almost 80% of anal cancers contain integrated HPV (23). In the case of HPV-positive oropharyngeal squamous cell carcinomas, the incidence of viral integration is relatively lower, with many tumors having either extrachromosomal or mixed extrachromosomal and integrated viral DNA (24–26).

It has been suggested that DNA damage plays an essential role in HPV integration, since the process of integration requires linearization of the viral genome, breakage of the host genome, and insertion of viral genome followed by re-ligation of the ends together (27–30). Determining which agents and events are likely to cause breaks in the viral and host DNA is therefore a reasonable approach to identify the factors that impact HPV integration frequency. One of the most common agents known to cause DNA damage are reactive oxygen species (ROS), generated in excessive amounts under conditions of oxidative stress (31–33). Our lab has previously demonstrated a high variability in the background levels of oxidative stress markers between non-cancerous cervical cells and tissues of different women, which provides a possible explanation for why some, but not most, HPV-infected individuals seem predisposed to develop cervical cancer (34). Importantly, we have also shown that chronic oxidative stress, caused by environmental or viral factors such as E6*, can induce DNA damage and increase the frequency of integration of HPV16 into the genome of cervical keratinocytes, and in that way contribute to HPV-mediated carcinogenesis (35). Supporting our data, environmental conditions known to cause oxidative stress, such as smoking and co-infection with STD-associated pathogens *Chlamydia trachomatis* and *Neisseria gonorrhoeae*, have been associated with increased incidence of HPV-mediated malignancies (36–38), and several publications have reported increased oxidative stress in patients with HPV-related cancers (39–42).

Another potentially significant source of DNA damage is exposure to elevated doses and different qualities of ionizing radiation. Natural background radiation is ubiquitous; hence, all are exposed. However, some populations, including radiological workers, military personnel, radiation therapy patients, commercial pilots, and astronauts, are exposed to significantly higher acute or chronic doses of ionizing radiation (43). One type of ionizing radiation is proton radiation which is characterized by its physical properties as a subatomic particle with a mass and a positive charge that can be accelerated naturally in space (by the sun) or artificially (by particle accelerators).

In space, proton radiation is part of the radiation environmental spectra and can pose a health risk for humans during long- and short-term spaceflights (44, 45). Solar particle events (SPEs) which accelerate protons and release unpredictable doses of radiation is one of the space radiation environment components. SPE radiation exposure poses a threat to astronauts in a spacecraft where shielding is available, and especially during an extravehicular activity (EVA), in which shielding is limited. SPE radiation consists predominantly of energetic proton particles with energies greater than 10 MeV. It has been estimated that the largest dose of SPE radiation recorded from a historically large SPE (in August 1972) was capable of delivering a 32.4 Gy dose to the skin, 1.38 Gy blood forming organs (BFO) dose and 0.42 Gy to the stomach (during an EVA), and a 2.7 Gy to the skin, 0.46 Gy BFO dose and 0.17 to the stomach (inside spacecraft) to the astronauts (46).

On the other hand, on Earth, we artificially exploit proton’s characteristics to treat cancer. As a charged particle, protons can penetrate a certain depth in tissues depending on the energy of the proton (>150 MeV). Proton has physical advantages over photon radiation (X-rays, gamma rays) by depositing most of its energy at the Bragg Peak, beyond which there is no energy/dose delivered (47). Therefore, normal tissues distal to the Bragg peak can be protected by averting radiation doses. However, considerable dose or radiation (>1 Gy) are still delivered in the plateau region of the Bragg curve in front of the tumor volume. Overall, comparing to the most advanced photon techniques such as intensity-modulated radiotherapy (IMRT), proton therapy can deliver similar or higher radiation doses to tumor volumes with a reduction in integral radiation dose (50%–60% reduction) (48, 49). With the development of pencil beam scanning technique, intensity-modulated proton therapy (IMPT) can also be performed which yields highly conformal dose distribution around the target volumes (50). Because of these advantages, proton therapy has become the preferred radiotherapy modality for pediatric cancer patients and head and neck cancers as well as other tumor types in adults.

Ionizing radiation can induce DNA damage by its direct interaction with the target macromolecule, resulting in the disruption of the molecular structure. Additionally, it can act indirectly through radiolysis of water molecules, generating ROS particles that cause oxidative damage to the DNA (51, 52). The ability of radiation-induced DNA damage to cause genomic instability and cancer is well established (53). In addition, the use of conventional radiation to induce DNA damage and enhance DNA integration has been explored previously (54–57). However, little is known about the effect of particle radiation-induced DNA damage on HPV integration enhancement, an event that is critical for HPV carcinogenesis. We thereby postulate that the impact of proton-induced DNA damage is amplified in the case of oncogenic DNA viruses such as HPV, because such damage has the additional effect of increasing HPV integration frequency. In particular, we predict that radiation-induced damage to the host DNA puts the exposed population at higher risk of developing HPV-related cancers by increasing the likelihood of HPV integration. To assess this possibility, we exposed oral keratinocytes to protons, as a source of ionizing radiation, and demonstrated that proton-induced DNA damage promotes the integration of foreign DNA into the host genome. Establishing that protons exposure increases the process of HPV integration enables the development of strategies designed specifically to prevent such integration in radiation-exposed populations, with an ultimate goal of reducing or eliminating these malignancies.

## Materials and methods

### Cell culture

Normal oral keratinocytes (NOK), non-transformed cells immortalized by Human Telomerase

Reverse Transcriptase (hTERT) that were kindly provided by Dr. Karl Münger (58), were grown in keratinocyte serum-free medium (Invitrogen, Carlsbad, CA), supplemented with penicillin (100 U/ml) and streptomycin (100 μg/ml) (Sigma-Aldrich, St. Louis, MO).

### Exposure to protons

Cell irradiation with protons was completed at the James M. Slater Proton Treatment and Research Center, Loma Linda California. Exposures were done at room temperature. Two hundred and fifty MeV protons were modulated to generate a 5.0 cm wide spread-out Bragg peak (SOBP). The cells were located at a water equivalent depth of 29.6 cm, specified using CIRS plastic water blocks, which placed the cells in the uniform dose SOBP region of the proton dose profile. Irradiations were conducted with the beam incident on the underside of the flask to ensure accurate placement of the cell layer with respect to the proton depth dose profile. The proton field size employed for the irradiation of the NOK cells was circular with an 18 cm diameter. Protons were delivered from our synchrotron accelerator in a pulsed fashion, with a pulse duration of 0.125 seconds and a duty cycle of 2.2 seconds. This pulsed modality of beam delivery gave a dose rate of approximately 0.8 Gy/min. The integrated number of protons per spill or per exposure is measured by the transmission ionization chamber (TIC) and a secondary electron emission monitor. The beam position and beam profile are monitored using multiwire ion chambers (MWICs). The wire chamber resolution is 2 mm. A 25 × 25 cm^2^ ion chamber segmented into 400 square pads are placed after the range modulator to monitor the dose delivered to the target volume. The detector consists of a 20 × 20 array of pads (each 1.25 × 1.25 cm^2^) from a thin sheet of gold-plated Kapton. Any of the pads in the central region of the pad plane can be used to monitor and control the dose delivered to the target and the remaining pads are also used to study the transverse dose distribution (59). Depending on the experimental setting, cells were exposed to single doses of 0, 0.5, 1, 2, 4, 8 Gy.

### γ-H2AX foci formation using immunofluorescence

Double-strand DNA breaks and the subsequent repair of DNA lesions were monitored by examining the formation of γ-H2AX *foci* using antibodies specific for the phosphorylation of Ser-139 at the C-terminal region. Thirty thousand cells were seeded into each well of 4-well chamber slides. After treatment, cells were fixed in methanol for 15 min, washed with PBS, and permeabilized in 0.5% Triton X-100 for 5 min. After blocking with blocking serum (5% goat serum-0.3% Triton X-100) for 30 min, cells were stained with anti-γH2AX mouse monoclonal antibody (1:200 dilution; Abcam, Cambridge, MA) for 1 h, and AlexaFluor-488 goat anti-mouse secondary antibody (1:600 dilution; Invitrogen, Carlsbad, CA) for another hour. Coverslips were mounted with Vectashield Mounting Medium containing DAPI (Vector Laboratories, Burlingame, CA), to counterstain cellular nuclei. The slides were observed with an Olympus BX51 fluorescence microscope and images were taken using Olympus CellSens Standard software.

### Measurement of reactive oxygen species using dichlorodihydrofluorescin DiOxyQ

Two and a half million cells were centrifuged at 2000 rpm (450 xg) for 5 minutes and washed with 1 mL PBS. The pellet was resuspended in 500 µl PBS and homogenized for 2 minutes. The sample was then centrifuged at 10,000 rpm at 4°C for 5 minutes, and the supernatant was aliquoted in an eppendorf tube and stored at -80°C until use. The levels of reactive oxygen and nitrogen species (i.e. hydrogen peroxide, peroxyl radical, nitric oxide and peroxynitrite anion), generally referred to as “ROS”, were then measured in these cell homogenates using the OxiSelect *In Vitro* ROS/RNS assay kit (Cell BioLabs, San Diego, CA). The fluorescence intensity was detected at 480 nm excitation/530 nm emission using a SpectraMax i3X fluorometric plate reader (Molecular Devices). Protein concentrations were also measured with the Coomassie Plus Assay Reagent (Thermo Fisher Scientific, Rockford, IL) and were used for normalization. Therefore, ROS levels are expressed as Relative Fluorescence Unit (RFU) per μg of protein.

### Assessment of 8-oxoG DNA damage using avidin-FITC

Oxidative DNA damage was determined *via* the direct binding of fluorescein isothiocyanate (FITC)-labeled avidin to 8-hydroxy-2’-deoxyguanosine (8-oxoG) residues in the genomic DNA. Briefly, 5 x 10^6^ cells were collected, washed twice with PBS, and fixed with 4% paraformaldehyde for 15 minutes. Cells were then washed three times with PBS, permeabilized with 75% ethanol and stored at -20°C until ready for experimental use. All samples were washed, blocked, and incubated with 2 µg/ml Avidin-FITC (Thermo Fisher Scientific, Rockford, IL) for 1 h in the dark. After two washes, they were resuspended in PBS and analyzed by flow cytometry for fluorescence (excitation 495 nm, emission 515 nm) on a MACSQuant Analyzer 10 (Miltenyi Biotec Inc). A total of 10,000 events were measured per sample and data were analyzed using FlowJo software.

### Trypan blue exclusion test

The short-term cytotoxicity of proton exposure was examined using the trypan blue exclusion test. While viable cells remain unstained, those with damaged membrane stain blue. Radiation-exposed cells, collected 48-72 hours after radiation exposure by trypsinization, were re-suspended in 1 ml of media, mixed with 0.4% trypan blue solution at a ratio of 1:1, and counted using a Biorad TC20 automated cell counter. The ratio of unstained cells to the total number of cells (stained and unstained) was used to determine the percentage of cell viability, while the total cell density relative to the control group was used to estimate the rate of cell proliferation.

### Colony formation assay

The long-term cytotoxicity of proton exposure was determined using a clonogenic assay. Cells were trypsinized and seeded in 6-well plates at specific numbers (100 cells for 0 Gy; 200 cells for 1 Gy; 300 cells for 2 Gy; 400 cells for 4 Gy; and 800 cells for 8 Gy). After an overnight incubation, the cells were subjected to different doses of radiation. The cells were incubated at 37 °C for an additional 8 to 10 days, fixed with methanol:acetic acid (3:1 ratio) for 5 min, and stained with 1% crystal violet for 1 h. Plates were then rinsed with water and left to dry overnight at room temperature. Colonies with >50 cells were counted using ImageJ. Plating efficiency and surviving fraction for given treatments were calculated based on the survival of non-treated cells (60, 61).

### Cell cycle analysis

The effect of radiation exposure on cell cycle progression was examined using propidium iodide (PI) DNA staining. The fluorescence intensity of PI-stained DNA reflects the DNA content of a cell, which determines the proportion of cells in the different cell cycle phases. Forty-eight to seventy-two hours after radiation exposure, 3 x 10^6^ cells were washed with PBS, and fixed in ice cold 70% ethanol for at least 1h at -20°C. They were then allowed to warm at room temperature, washed with PBS, and resuspended in 0.5 mL of FxCycle PI/RNase A staining solution (Thermo Fisher Scientific, Rockford, IL) for 30 min, following which PI-elicited fluorescence was measured using the MACSQuant Analyzer 10 (Miltenyi Biotec Inc). Each sample was collected as 10,000 events, and the corresponding cell cycle distribution was then determined according to the DNA content on FlowJo software.

### Apoptosis detection using annexin V/7AAD staining

Apoptosis was assessed using the Annexin V Apoptosis Detection Kit with 7AAD (BioLegend, San Diego, CA). Forty-eight to seventy-two hours after radiation exposure, 2 x 10^5^ cells were added in triplicate into a 96 well plate, rinsed with PBS, re-suspended in 10 µl of 1X annexin V binding buffer, and labeled with 1 µl of Annexin V for 15 min. 1 µl of 7AAD was then added for 5 min for dead cell discrimination. Finally, 180 µl was added to Annexin V binding buffer, following which samples were analyzed using the MACSQuant Analyzer 10 (Miltenyi Biotec Inc). Annexin V-/7AAD− cell population was considered healthy, Annexin V-/7AAD+ was indicative of necrotic cells, whereas the Annexin V+/7AAD− and Annexin V+/7AAD+ were representative of early and late apoptotic cells, respectively.

### Puromycin-resistance clonogenic assay in NOK cells

Five hundred thousand cells were seeded in 6-well plates, and 24 hr later exposed to radiation, following which they were transfected with pMetluc *puro* plasmid encoding the puromycin resistance gene. 48 hours later, the media was collected and the expression of secreted *Metridia* luciferase was measured using the ready-to-glow secreted luciferase reported assay (Takara Bio USA Inc) for normalization of the transfection efficiency. The integration frequency of this foreign circular DNA was then assessed using a clonogenic assay as previously described (35). Briefly, selection of cells resistant to puromycin at a concentration of 5 μg/ml was performed for 2-3 weeks. After antibiotic selection, the resulting colonies were fixed using 10% formaldehyde for 30-45 min. After rinsing with water and drying overnight at room temperature, cells were stained with 1% crystal violet for 1 hour. Plates were then rinsed with water and left to dry overnight at room temperature. Colonies were counted the following day and normalized for transfection efficiency by MetLuc activity. Integration frequency was compared between untreated cells and cells treated with the indicated doses of radiation.

### Statistical analysis

Statistical significances were determined using the SPSS software. Student’s *t* test was applied and a p-value < 0.05 was regarded as significant. For each parameter tested, a set of three different experiments were performed. Data are represented as the mean ± standard error of the mean.

## Results

### Proton radiation induces dsDNA breaks promptly and dose-dependently in NOK cells

**Figure 1:**
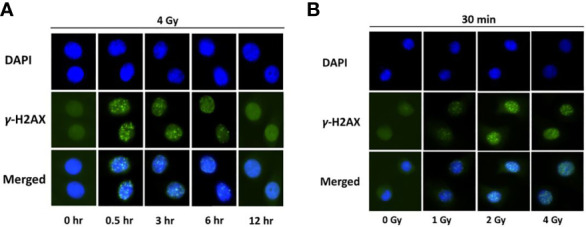
H2AX is phosphorylated in normal oral keratinocytes exposed to proton radiation. **(A)** Immunofluorescence of γ-H2AX at different time-points (0, 0.5, 3, 6, 12 hr) in normal oral keratinocytes irradiated with 4 Gy of protons. Nuclear DNA was counterstained with DAPI. **(B)** Immunofluorescence of γ-H2AX in NOK cells, 30 minutes after exposure to different doses of proton radiation (0, 1, 2, 4 Gy). Nuclear DNA was counterstained with DAPI. Images are representative of three independent experiments.

Radiation-induced DNA damage can be caused by its direct interaction with the DNA, thereby disrupting the molecular structure. To examine the kinetics of direct DNA damage following proton exposure, NOK cells were irradiated with a high dose of protons (4 Gy), fixed at different time-points (0, 0.5, 3, 6, 12 hr), and stained with γ-H2AX antibodies (**Figure 1A**). Our results show that foci formation, indicative of double-stranded DNA breaks, is at its highest intensity at 0.5 hr, and continuously decreases with time to be almost undetected at 12 hr.

After detecting the time-point with the highest signal of γ-H2AX (0.5 hr), we examined the dose-dependent effect of proton exposure on direct DNA damage. NOK cells were treated with different doses of proton (0, 1, 2, and 4 Gy), and 30 min later were fixed and stained with γ-H2AX (**Figure 1B**). As expected, foci formation increased dose-dependently following exposure with different doses of ionizing radiation.

### ROS-induced DNA damage is delayed but occurs dose-dependently in proton-exposed NOK cells

**Figure 2:**
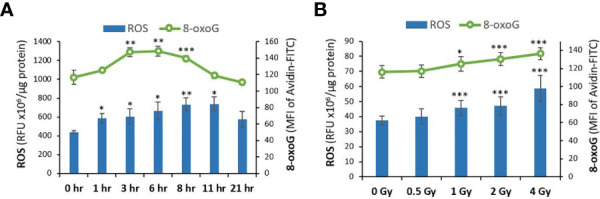
ROS and oxidative DNA damage levels are increased in normal oral keratinocytes exposed to proton radiation. **(A)** The levels of ROS and the resulting oxidative DNA damage biomarker 8-oxoG were analyzed using DCF and Avidin-FITC staining in NOK cells irradiated with 4 Gy of protons at different time-points (0, 1, 3, 6, 8, 11, 21 hr). **(B)** ROS and 8-oxoG were measured in NOK cells irradiated with different doses of proton radiation (0, 1, 2, 4 Gy) 6-8 hours after exposure. Asterisks on bars represent significance relative to the control group. (*), (**), and (***) correspond to p<0.05, 0.01, and 0.001, respectively.

Radiation exposure can also act indirectly through radiolysis of water molecules, generating ROS particles that cause oxidative damage to the DNA. The kinetics of indirect DNA damage following proton exposure was assessed by measuring the levels of ROS and the resulting oxidative DNA damage 8-oxoG at different time-points (0, 1, 3, 6, 8, 11, 21 hr) in NOK cells irradiated with 4 Gy of protons (**Figure 2A**). As expected, indirect DNA damage was delayed due to the time needed for ROS particles to accumulate and cause oxidative DNA damage. An increase in ROS levels was detected as early as 1 hr and increased with time up until 11 hours, whereas oxidative DNA damage was observed at only 3, 6, 8 hrs.

Based on these results, 6-8 hr post-exposure was selected as the time-point to examine the dose-dependent effect of protons on ROS-induced DNA damage. **Figure 2B** represents the measured levels of ROS and 8-oxoG in NOK cells irradiated with different doses of proton (0, 0.5, 1, 2, 4 Gy). Our results show that indirect DNA damage, similar to direct DNA damage, also increases dose-dependently following exposure to 1-4 Gy of proton radiation.

### Proton radiation is not cytotoxic at low doses, but is lethal at 8 Gy

**Figure 3:**
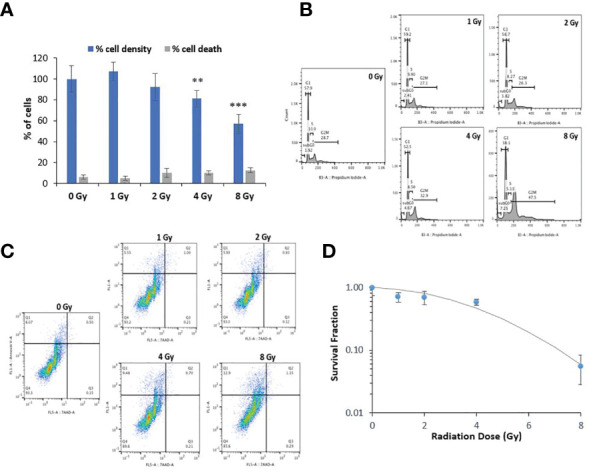
Proton radiation at 1-2 Gy does not cause significant toxicity but causes G2/M cell cycle arrest at higher doses. **(A)** Acute cytotoxic effect of proton exposure on NOK cells was assessed using the trypan blue exclusion test at 48-72 hr. The ratio of stained cells to the total number of cells was used to determine the percentage of cell death in each treated group (in gray), while the total cell density relative to the control group as 100% (in blue) was used to estimate the rate of cell proliferation. Asterisks on bars represent statistical significance in total cell density relative to control (** for p<0.01; *** for p<0.001). **(B)** Representative image of one of three experiments showing the cell cycle phases of NOK cells exposed to 0-8 Gy of protons at 48-72 hr. **(C)** Representative image of one of three experiments showing the Annexin V/7AAD profile in NOK cells exposed to 0-8 Gy of protons at 48-72 hr. Annexin V-/7AAD− cell population is considered healthy, Annexin V-/7AAD+ is indicative of necrotic cells, whereas the Annexin V+/7AAD− and Annexin V+/7AAD+ are representative of early and late apoptotic cells, respectively. **(D)** Analysis of cell survival fraction by colony formation assay in NOK cells treated with 0-8 Gy of protons 1-2 weeks after exposure.

Radiation exposure can be detrimental at high doses. Therefore, we examined the potential short- and long-term cytotoxicity of the selected doses of protons on normal oral keratinocytes. We assessed the acute cytotoxic effect of proton exposure on NOK cells at 48-72 hr using the trypan blue exclusion test (**Figure 3A**). Although the percentage of cell death in NOK cells exposed to 1-8 Gy of protons did not significantly increase in the treated groups (in gray), the cell density was significantly decreased at 4 Gy (81%) and 8 Gy (57%) relative to the control group (in blue). To confirm and further elucidate these results, we performed cell cycle analysis using propidium iodide and Annexin V apoptosis detection assay. Our cell cycle analysis (**Figure 3B**) showed no remarkable changes in cell cycle distribution of NOK cells exposed to 1 and 2 Gy. However, 4 and 8 Gy treatments resulted in a dose-dependent increase in G2/M phase (32.9% and 47.5%, respectively), providing an explanation for the decrease in cell density at these doses. A minimum dose-dependent increase in subG0 was also obtained in NOK cells exposed to 1-8 Gy, indicating a minor induction of cell death. On the other hand, our Annexin V/7AAD profile did not show any significant increase in the levels of apoptotic and necrotic cells in any of the treated cells, except for a slight increase in the number of Annexin V+/7AAD- pre-apoptotic cells in 4 Gy (9.48%) and 8 Gy (12.9%) (**Figure 3C**), validating our trypan blue assay and cell cycle analysis results.

Delayed toxicity (1-2 weeks) was also examined in irradiated NOK cells using the standard colony formation assay (**Figure 3D**). Consistent with the other cytotoxicity assays, 1 and 2 Gy did not cause any significant effect on the survival and ability of the cells to undergo “unlimited” division; 4 Gy showed some significant cytotoxicity, whereas 8 Gy was found to be highly lethal.

### Proton exposure induces integration of plasmid DNA into NOK cells

**Figure 4:**
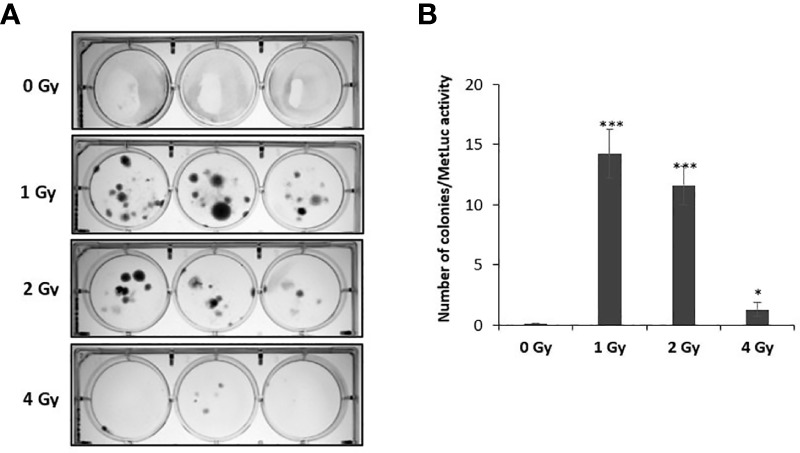
Exposing NOK cells to proton radiation results in increased frequency of pMetLuc plasmid integration. 5 × 10^5^ cells/well of NOK cells were exposed to proton radiation (0, 1, 2, 4 Gy), following which they were transfected with pMetluc *puro* plasmid. To normalize for transfection efficiency, the media was collected 48 hr post-transfection, and the expression of secreted luciferase was measured. Selection of cells resistant to puromycin (5 μg/ml) was performed daily for 2-3 weeks, following which the colonies produced were stained with crystal violet **(A)**, and normalized for transfection efficiency **(B)**. (*), and (***) correspond to p < 0.05, and 0.001, respectively.

To investigate whether exposure to proton radiation increases the rate at which circular plasmid DNA integrates into the human genome, we performed a clonogenic assay. NOK cells were irradiated with the selected doses (0, 1, 2, 4 Gy), following which they were transfected with pMetLuc plasmid encoding for puromycin resistance. To normalize for transfection efficiency, the expression of Metridia luciferase was monitored in the media. After puromycin selection for 2-3 weeks, the resistant colonies were stained with crystal violet and counted (**Figure 4A**). The number of colonies normalized to luciferase activity is presented in **Figure 4B**. As anticipated, no antibiotic resistant clones were observed in the untreated wells following selection, while numerous stable clones were noted in wells treated with 1 and 2 Gy, demonstrating that DNA damage induced by protons promotes the integration of plasmid DNA in NOK cells. On the other hand, 4 Gy-treated wells had relatively fewer number of clones likely due to the observed toxicity.

## Discussion

The ability of radiation to damage DNA, thereby leading to single or double-stranded breaks, larger-scale damage and cancer, is well established (53). In the study reported here, we hypothesize that the impact of proton-induced DNA damage is magnified in the case of oncogenic DNA viruses such as HPV, because such damage has the additional effect of increasing integration frequency and inducing carcinogenesis. Therefore, our overall goal was to explore the effect of DNA damage induced by ionizing radiation such as protons on the integration of foreign DNA into the human genome.

DNA is one of the critical targets of ionizing radiation. The most dangerous type of DNA lesion caused by ionizing radiation is the complete break of the DNA double helix, and one of the well-characterized markers of double stranded DNA breaks is the phosphorylation of the histone H2AX (γ-H2AX) by the ATM and/or DNA-PK kinases at dsDNA break sites. Therefore, formation of γ-H2AX foci indicates the presence of dsDNA breaks, while foci disappearance is associated with the repair of the damaged regions of the DNA. In this study, we first determined the kinetics of direct DNA damage caused by proton exposure in irradiated normal oral keratinocytes at different time-points. Our results show that histone H2AX is rapidly phosphorylated following radiation exposure, with a peak of H2AX phosphorylation at 30 minutes after irradiation, after which it continuously decreases with time to be almost undetectable by 12 hr (**Figure 1A**). These results are consistent with a study by Mariotti et al. (62), in which cells exposed to 1 and 2 Gy of X-ray reached the maximum number of γ-H2AX foci formation at 30 min after irradiation, after which the resulting foci kinetics followed a clear negative exponential response with very little residual damage detectable by 24 hr post exposure. After determining the kinetics of γ-H2AX foci formation in normal oral keratinocytes following proton exposure, we assessed the dose-response of proton exposure on direct DNA damage 30 min after exposure. As expected, foci formation was detected to be dose-dependently increased following exposure to 0-4 Gy of protons (**Figure 1B**), similar to previous reports in other cell lines exposed to X-ray and alpha-particles (63, 64).

When cells, which are composed of more than 70% of water, are exposed to ionizing radiation, radiolysis of water molecules occurs, resulting in the production of reactive oxygen species (ROS) and reactive nitrogen species (RNS). Persistent and excessive generation of ROS causes oxidative insults to cellular components including nucleic acids, proteins and lipids (65, 66). These oxidizing events induced by ionizing radiation can persist for days and months due to the continuous generation of ROS species. Furthermore, radiation-induced oxidative stress may spread from targeted cells to non-targeted bystander cells through intercellular communication mechanisms (51). Therefore, the molecular and biochemical events that promote oxidative stress in irradiated cells play crucial roles in mediating the harmful effects of ionizing radiation. From the many types of oxidative DNA damage products, 8-hydroxy-2’-deoxyguanosine, derived from hydroxyl radical attack of deoxyguanosine residues, is the most used and representative biomarker of oxidative DNA damage (67, 68). We therefore analyzed the levels of ROS and the resulting 8-oxoG hydroxylation product in irradiated cells to assess the effect of proton radiation on indirect ROS-induced DNA damage. As anticipated, indirect DNA damage was delayed for hours in NOK cells exposed to protons, due to the time required for ROS particles to build up and cause oxidative DNA damage (**Figure 2A**). An increase in ROS levels was detected as early as 1 hr post-exposure and was continuously increased to reach a plateau at 11 hr. It then decreased to be non-significant at 21 hr, likely due to the activation of antioxidant defensive mechanisms of the cells. These results are consistent with a study by Yamamori et al. (69) showing that X-ray irradiation induces a time-dependent increase in ROS levels, peaking at 12 hr, and declining by 24 hr. On the other hand, our results demonstrate that oxidative DNA damage is induced at 3 hr, reaches a peak at 6-8 hrs, and becomes completely repaired at 11 hr and 21 hr. Importantly, our oxidative DNA damage results are concomitant with those of ROS levels in irradiated NOK cells, in that ROS accumulation occurs first, which later leads to oxidative damage to the DNA. Finally, similar to our observations with direct DNA damage, we also demonstrated that indirect ROS-induced DNA damage increases dose-dependently following exposure of NOK cells to 1-4 Gy of protons for 6-8 hr (**Figure 2B**).

In response to DNA damage induced by ionizing radiation, numerous cellular signaling pathways are anticipated to be activated in irradiated cells, which have the potential to result in cell cycle checkpoint activation/DNA repair, apoptosis, and cellular senescence (70). To ensure that the proton doses used in our experiments do not cause major cytotoxic effects which could interfere with our clonogenic integration study, we carried out cytotoxicity assays. Acute cytotoxicity assessed by the trypan blue exclusion test showed a dose-dependent decrease in total cell density in NOK cells exposed to 4 and 8 Gy of protons compared to the untreated group, though no significant increase in the percentage of dead cells was noted at these doses (**Figure 3A**). The decrease in the cell density at 4 and 8 Gy is explained by the observed G2/M cell cycle arrest at these doses (**Figure 3B**). On the other hand, the Annexin-V/7AAD profile did not display an increase in the number of apoptotic or necrotic cells at any of the doses, except for a slight increase in the levels of pre-apoptotic cells at 4-8 Gy (**Figure 3C**), validating the limited number of dead cells noted in our trypan blue results. Together, the remarkable halt in cell cycle progression and the relative absence of apoptotic/necrotic cells point toward an effect of proton exposure on cell proliferation rather than on cell viability. In response to DNA damage, cell cycle progression often becomes blocked by the activation of cell cycle checkpoints, allowing the cells to repair the damage prior to replication. In cases where the damage is irreversible or the cell cycle checkpoint is dysfunctional, apoptosis may be triggered to eliminate the damaged cells (71). While the cell cycle arrest detected at 4 Gy seems to be temporary, the cells exposed to 8 Gy appears to be more permanent, as the results of colony formation assay show 4 Gy to be slightly cytotoxic, while 8 Gy to be highly lethal (**Figure 3D**). Interestingly, previous studies have reported that the mitochondrial content, mitochondrial electron transport chain function, and mitochondrial ROS production peak in the G2/M phase of the cell cycle (72, 73), and that accumulation of irradiated cells in G2/M phase under the control of G2/M checkpoint result in a corresponding increase in mitochondrial ROS level (69). Hence, it has been suggested that ROS can also be released from biological sources such as mitochondria in irradiated cells, in addition to its byproduct generation from radiolysis of water molecules. Our results showing proton exposure inducing excessive ROS generation may therefore be at least partly due to the ionizing radiation-induced cell cycle arrest at the G2/M phase.

Following our establishment of an experimental system in which proton exposure induces both types of DNA damage without causing major cytotoxicity, we explored the impact of high doses of proton radiation on the integration of foreign DNA, the pMetLuc plasmid, into the host genome using a clonogenic assay. This integration model is well established in our lab and has been previously employed to examine the effect of chronic oxidative stress on HPV integration frequency (35. The genetic material used in this system works as a good model to study HPV integration, since it shares many critical features with the HPV genome. They are both small in size (<8kb), circular, double stranded DNA, and most importantly, they rarely integrate into the host genome spontaneously. Consistent with our working model, we demonstrated that exposure to proton radiation induces the integration of HPV-like foreign DNA into the human genome (**Figures 4A, B**). These findings are in line with previous studies, in which various doses of γ- and X-ray ionizing radiation have been shown to stimulate integration of different DNA vectors into the host cell genome (54, 57, 74, 75). However, to the best of our knowledge, we are the first to explore the effect of high doses of protons on DNA integration, and particularly in the context of HPV. Therefore, our results add to the broad data on the deleterious effects of ionizing radiation and allow us to predict that proton-induced damage to the host DNA puts the exposed population at higher risk of developing HPV-related cancers by stimulating HPV integration. The concept of ionizing radiation inducing DNA integration has also been explored as a tool to enhance DNA-mediated gene transfer in mammalian cells (55, 56, 76, 77). Administering radiation prior to adenovirus-mediated gene therapy holds promise to greatly improve the adenoviral transduction efficiency and overcome the low rate of stable gene transfer. However, this combined modality of radiation-guided gene therapy should be considered with extra caution since it could result in unintentional integrations of oncogenic viruses such as HPV, putting infected individuals at higher risks for oncovirus-mediated carcinogenesis.

It is well established that exposure to elevated doses of ionizing radiation contribute to carcinogenesis. The carcinogenic potential of ionizing radiation was recognized shortly after Roentgen’s discovery of X-rays, when the first radiation-induced skin cancer was reported in 1902. Ever since, several experimental studies have identified the general characteristics of radiation carcinogenesis, and various epidemiological studies in human populations exposed to occupational, medical, and accidental sources of radiation have supported the emerging findings (78). Radiation can induce a broad spectrum of DNA lesions including damage to nucleotide bases, cross-linking and DNA single- and double-strand breaks, with the latter being most important type of biological lesion (79, 80). Most of these dsDNA breaks are repaired by the error prone non-homologous end joining (NHEJ) or microhomology-mediated end joining (MHEJ) mechanisms, and thereby facilitate the generation of gene mutations, chromosomal abnormalities and other large-scale changes that are frequently detected in irradiated cells (81–85). While radiation is considered to be a relatively weaker carcinogen and mutagen compared to certain chemicals such as the polycyclic hydrocarbons, various secondary factors can modulate its hazardous effects and contribute to cancer development (78). In this study, we report for the first time that proton-induced damage to DNA stimulates integration of foreign DNA into the human genome, amplifying the carcinogenic potential of oncogenic viruses such as HPV.

Integration of the viral genome into the host genome is a pivotal step in the process of malignant transformation by several oncogenic viruses, including Hepatitis B virus (HBV), Merkel cell polyomavirus (MCV) and HPV [reviewed in (30)]. These viruses do not encode genes that produce integrase enzymatic activity protein; therefore, the integrative process of these oncogenic viruses is likely mediated by cellular genes involved in DNA replication and repair mechanisms (86–88). Since viral integration requires breakage of both the viral and the host DNA, the likelihood of integration depends on the levels of DNA damage. In support of this idea, several pieces of evidence have linked oxidative DNA damage and dsDNA breaks to a higher integration frequency of HBV (89–91). Furthermore, following dsDNA breaks, the recruitment of DNA damage repair complexes ensures the accessibility of ligases that can reconnect the recombined host and viral sequences, creating the perfect microenvironment for viral integration. In the case of HPV, the homologous recombination process is unlikely to contribute to HPV integration, as there is insufficient homology between HPV sequences and the human genome. On the other hand, microhomology-mediated DNA repair pathways have been found to be involved in the ligation of linearized HPV16 and host DNA, as evidenced by enriched micro-homologous sequences between HPV and human genomes at the integration breakpoints in both cervical and oropharyngeal cancers (25, 92–94). This integration model of HPV is consistent with our working model that radiation-induced dsDNA breaks activate DNA repair pathways mediated by end joining which would in its turn facilitate the integration of HPV into the host genome.

## Conclusions

We determined the kinetics of DNA damage in normal oral keratinocytes exposed to proton radiation by assessing *γ-*H2AX foci formation using immunofluorescence (direct damage) and measuring ROS and 8-oxoG levels *via* DCFDA and Avidin-FITC (indirect damage). As anticipated, direct DNA damage was observed promptly, within 30 min, whereas indirect DNA damage was delayed due to the time required for ROS to accumulate and cause oxidative damage. Although protons were toxic at high doses (4-8 Gy), we were able to establish an experimental system where exposure to proton radiation induced DNA damage dose-dependently without causing major cytotoxic effects as assessed by several cytotoxicity assays. Most importantly, we explored the impact of proton exposure on integration frequency using a clonogenic assay, and showed that as predicted, DNA damage induced by proton exposure promotes the integration of foreign DNA in oral keratinocytes, increasing the risks of HPV malignancies. Our results demonstrating the amplified impact of proton-induced DNA damage in the case of oncogenic DNA viruses such as HPV accentuate the deleterious effects of ionizing radiation and shed light on the extra precautions that need to be taken particularly in HPV-infected populations. Overall, the insights gained from this work enable us to better understand the contribution of proton exposure and DNA damage to HPV integration and risk of subsequent carcinogenesis and direct us toward strategies aimed at preventing malignancies in HPV-infected individuals.

## Data availability statement

The original contributions presented in the study are included in the article/supplementary materials, further inquiries can be directed to the corresponding author/s.

## Author contributions

MK performed the experimental work and data analysis and drafted this manuscript. AB and MV carried out the ionizing radiation exposures of the cells and contributed to the overall study design. VF participated in developing the overall concepts. XC helped with insightful discussion regarding DNA integration. PD-H was responsible for the overall study design and manuscript finalization. All authors contributed to the article and approved the submitted version.

## Funding

Funding for this study was provided by Loma Linda University (LLU). Other than the clear contributions by the listed LLU-affiliated authors, the funding body had no role in the design of the study and collection, analysis, and interpretation of data or in writing the manuscript.

## Acknowledgments

We thank Dr. Charles Wang and Dr. Isaac Kremsky for helpful and critical discussions of this work. We also thank to Dr. Jerry D. Slater for providing access to the proton facilities and supporting laboratories.

## Conflict of interest

The authors declare that the research was conducted in the absence of any commercial or financial relationships that could be construed as a potential conflict of interest.

## Publisher’s note

All claims expressed in this article are solely those of the authors and do not necessarily represent those of their affiliated organizations, or those of the publisher, the editors and the reviewers. Any product that may be evaluated in this article, or claim that may be made by its manufacturer, is not guaranteed or endorsed by the publisher.
